# Adhesive hydrogel wrap loaded with Netrin-1-modified adipose-derived stem cells: An effective approach against periarterial inflammation after endovascular intervention

**DOI:** 10.3389/fbioe.2022.944435

**Published:** 2022-07-22

**Authors:** Yihong Jiang, Yuting Cai, Jiateng Hu, Xing Zhang, Jiahao Lei, Zhaoxi Peng, Qun Huang, Zhijue Xu, Bo Li, Jinbao Qin, Weimin Li, Dazhi Sun, Kaichuang Ye, Xinwu Lu

**Affiliations:** ^1^ Department of Vascular Surgery, Shanghai Ninth People’s Hospital, Shanghai Jiao Tong University School of Medicine, Shanghai, China; ^2^ Department of Chemical and Biological Engineering, William Mong Institute of Nano Science and Technology, The Hong Kong University of Science and Technology, Kowloon, Hong Kong SAR, China; ^3^ Guangdong Provincial Key Laboratory of Functional Oxide Materials and Devices, Southern University of Science and Technology, Shenzhen, Guangdong, China

**Keywords:** endovascular interventions, Netrin-1, adipose-derived stem cells, macrophages, intimal hyperplasia, re-endothelialization, adhesive hydrogel, dopamine

## Abstract

Endovascular interventions, such as balloon dilation and stent implantation, are currently recommended as the primary treatment for patients with peripheral artery disease (PAD), greatly improving patient prognosis. However, the consequent lumen restenosis that occurs after endovascular interventions has become an important clinical problem. Inflammation has been proven to be crucial to postoperative restenosis. In previous studies we have identified that Netrin-1-modified adipose-derived stem cells (N-ADSCs) transplantation is an effective anti-inflammatory strategy to repair vascular damage. Nevertheless, it remained to be explored how one could constantly deliver N-ADSCs onto damaged arteries. Therefore, we developed an adhesive double network (DN) hydrogel wrap loaded with N-ADSCs for sustained perivascular delivery. Inspired by the adhesion mechanism of mussels, we developed an adhesive and tough polyacrylamide/calcium-alginate/reduced graphene oxide/polydopamine (PAM/CA/rGO/PDA) hydrogel. Dopamine was attached to graphene sheets and limitedly oxidized to generate free catechol groups. The hydrogel could wrap damaged arteries and induce anti-inflammatory effects through N-ADSCs. *In vitro* experiments demonstrated that N-ADSCs significantly promoted the M2 polarization of macrophages to anti-inflammatory phenotypes and reduced the expression of inflammatory factors. *In vivo* experiments in a rat carotid artery guidewire injury model showed that the adhesive hydrogel wrap loaded with N-ADSCs could significantly reduce arterial inflammation, inhibit intimal hyperplasia and improve re-endothelialization. Altogether, this newly developed N-ADSCs-loaded hydrogel wrap provides an effective slow-releasing system, which may be a promising way to prevent and treat restenosis after endovascular interventions.

## Introduction

Peripheral artery disease (PAD) is an ischemic disease caused by peripheral atherosclerotic stenosis and occlusion, which affects 230 million individuals worldwide. Up to 11% of PAD patients develop chronic limb-threatening ischemia (CLTI) with high rates of amputation and death. Endovascular interventions have gradually replaced open surgeries in the treatment of PAD. Indeed, the 2020 Global Vascular Guidelines recommended endovascular interventions (e.g., balloon dilation, stent implantation) as the first line of treatment for CLTI patients (Conte et al., 2019). However, endovascular interventions lead to a risk of up to 55% of postoperative vascular lumen restenosis. Although new drug-coated balloons and stents are currently being developed and implemented, the long-term restenosis rate is still 10%–20% (Razavi et al., 2018).

The pathophysiological mechanism that concurs lumen restenosis involves endothelial cell injury and intimal hyperplasia caused by chronic inflammation. Endovascular interventions inevitably result in mechanical injury of endothelial cells, impaired endothelial integrity, and endothelial cell dysfunction. These alterations lead to macrophage infiltration and migration of medial smooth muscle cells to the intima, culminating in lumen restenosis (Yahagi et al., 2014). Devices coated with paclitaxel or sirolimus were shown to inhibit smooth muscle cell proliferation as well as endothelial cell regeneration (Krankenberg et al., 2015). Therefore, the inhibition of inflammatory macrophage responses and intimal hyperplasia, coupled with the promotion of re-endothelialization are key to reduce postoperative lumen restenosis.

As innate immune cells, macrophages are the main inflammatory cells responsible for luminal restenosis (Sinha et al., 2021). Macrophages have remarkable plasticity and are mainly activated into two polarization phenotypes upon environmental stimuli. M1 macrophages are pro-inflammatory, thus aggravating inflammatory responses, while M2 macrophages are anti-inflammatory, thus inhibiting M1 macrophages, clearing apoptotic cells, and promoting tissue repair (Tabas and Lichtman, 2017). Previous studies have shown that after endovascular interventions, M1 macrophage infiltration and the consequent inflammatory response are directly related to vascular intimal hyperplasia (Kamann et al., 2019). Meanwhile, M2 macrophages can inhibit the phenotypic transformation of smooth muscle cells and reduce intimal hyperplasia (Yan et al., 2020). Our previous study has shown that depletion of macrophages can partially reduce luminal restenosis (Zhang et al., 2019), however this also eliminates beneficial M2 macrophages. Therefore, novel strategies are needed to inhibit M1 and promote M2 macrophages.

Given the rapid development of regenerative medicine and stem cell transplantation, various stem cells have been used in bioengineering protocols. Adipose-derived stem cells (ADSCs) have a high proliferation rate and multi-directional differentiation potential. Previous studies have found that transplanted ADSCs induce paracrine effects rather than differentiating into a specific cell type (Gnecchi et al., 2016). ADSCs secrete large amounts of exosomes, beneficial growth factors and cytokines to accelerate tissue repair (Adamiak et al., 2018). Indeed, ADSC exosomes and their conditional medium can induce macrophage polarization to the M2 phenotype to protect nerves and blood vessels from inflammation and help tissue regeneration and repair (He et al., 2019).

Netrin-1 was the first identified axon guidance factor. Netrin-1 and G-netrin share homology to the laminin gamma chain since they have a repeating 3 laminar epidermal growth factor (V-1, V-2, V-3) and a carboxy-terminal region. Studies have demonstrated that Netrin-1 promotes neuronal migration and secretion, and regulates endothelial and stem cell survival, adhesion, migration, proliferation and differentiation (Ding et al., 2014). Recent studies have found that Netrin-1 can inhibit the migration and chemokine production of inflammatory cells (Xia et al., 2022). Netrin-1 was reported to induce M2-type polarization of macrophages, thereby reducing inflammation and reducing atherosclerosis (Ranganathan et al., 2013). Our previous study showed that ADSCs overexpressing Netrin-1 (N-ADSCs) secreted abundant Netrin-1 protein and various beneficial cytokines after *in vivo* transplantation, which significantly promoted angiogenesis and muscle regeneration in diabetic denervated mice (Zhang et al., 2018). However, novel strategies are needed to continuously release Netrin-1 and cytokines *in vivo.* Recently, hydrogels have become a promising tissue engineering material that can mimic the natural cellular environment (Zhu and Marchant, 2011). With 3D networks and high porosity, hydrogels can achieve high loading of drugs and cells for sustained delivery, which in turn leads to promising therapeutic results (Vo et al., 2012). Compared with the delivery of single network (SN) hydrogels, double network (DN) hydrogels can protect cells from mechanical damage and promote cell retention *in vivo* (Gu et al., 2018). Meanwhile, adhesiveness is a vital characteristic of hydrogels that improve tissue repair, since this material can tightly stick to damaged tissues (Ghandforoushan et al., 2022). Based on the adhesive mechanism of mussels, highly adhesive hydrogels were fabricated by introducing catechol groups of polydopamine (PDA) (Han et al., 2017). In addition, graphene oxide showed potential for accelerating stem cell adhesion, proliferation, and differentiation (Lee et al., 2011).

Therefore, in the present study we designed a polyacrylamide/calcium-alginate/reduced graphene oxide/polydopamine (PAM/CA/rGO/PDA) hydrogel to treat PAD rats subjected to endovascular interventions. These hydrogels were wrapped around the artery and injected with N-ADSCs, so that the retained N-ADSCs could sustainably and effectively release Netrin-1 and beneficial cytokines. Such sustained release would induce the M2 polarization of perivascular macrophages, thereby reducing inflammation, inhibiting intimal hyperplasia and promoting re-endothelization. These newly developed hydrogels are a promising strategy to improve the clinical treatment of lumen restenosis after endovascular interventions.

## Materials and methods

### Synthesis of graphene oxide

The GO solution was synthesized as described previously (Zhuang et al., 2016). In brief, 1 g ground expandable graphite was dissolved into 150 ml concentrated sulfuric acid in a flask. Then, 40 g KMnO_4_ was gradually added under strong stirring overnight. The flask was transferred to an oil bath heated to 60°C and maintained for 6 h with strong mechanical stirring. Afterward, the mixture was poured into 1 L of Deionized (DI) water followed by 40 ml of 30% H_2_O_2_. The GO sheets were purified by dialysis until the pH was around 7. The solution was lyophilized for 3 days to acquire a brown foam and stored at −4°C until further use.

### Preparation of polydopamine-capped reduced graphene oxide (rGO/PDA)

A total of 50 mg of GO foam and 50 mg of dopamine hydrochloride were dissolved into 150 ml of Tris-Cl buffer solution (pH = 8.5) with vigorous stirring at 60°C for 24 h rGO/PDA was collected after centrifugation and washed using DI water three times. The solution was lyophilized for 3 days, generating a black foam and stored at −4°C until further use.

### Synthesis of the adhesive double network hydrogel

1.5 g sodium alginate (SA) and 0.2 g ionic cross-linker CaSO_4_, were dissolved in 100 ml DI water and stirred overnight until a clear solution was obtained. Then, 2 g acrylamide (AM) and rGO/PDA foam were dissolved into 10 ml of the above solution together with 0.01 g N,N-methylenebisacrylamide and 0.02 g ammonium persulfate. This solution was stirred until homogeneous. Finally, 10 µl tetramethylethylenediamine (TEMED), was added for polymerization. The hydrogels were sealed for 24 h to avoid water loss.

### Characterization and performance of the adhesive double network hydrogel

Scanning electron microscopy (SEM, JEOL JSM-6390F) was used to characterize the morphology and microstructures of the adhesive hydrogel. The chemical composition of rGO/PDA hydrogels was confirmed by Fourier transform infrared 371 spectroscopy (FTIR; Vertex 70 Hyperion 1000). The mechanical property was measured by testing equipment (MTS Alliance RT-5) at a constant speed of 30 mm min^−1^ for strain loading.

### Tissue adhesive strength

The tissue adhesive strength of hydrogels was investigated by using a universal testing machine (MTS Alliance RT-5). Fresh porcine skins were purchased from the supermarket and cleaned using tissue paper. The adhesive hydrogel was applied onto porcine skins and pressed using two glass sheets for 1 min. The sizes of contact areas were around 1 cm*1 cm. The adhesive strength was measured by equipping the UTM with a load cell of 100 N at a speed of 30 mm min^−1^. Each experiment was repeated three times.

### Animals

Ten-week-old Sprague Dawley (SD) rats fed with a high-fat diet (21% fat, 0.15% cholesterol) were purchased from the laboratory animal center of Shanghai Ninth People’s Hospital, Shanghai Jiao Tong University School of Medicine. Animal procedures were performed following the Guidelines for Care and Use of Laboratory Animals of Shanghai Jiao Tong University School of Medicine with the approval of the Animal Ethics Committee of Shanghai Ninth People’s Hospital, Shanghai Jiao Tong University School of Medicine.

### Isolation, culture, and identification of adipose-derived stem cells

The adipose tissue of the axilla and inguinal region of 10-week-old SD rats was isolated and ADSCs were obtained as previously described (Bhattacharjee et al., 2022). ADSCs were incubated with low glucose (5.5 mM) Dulbecco’s modified Eagle’s medium (DMEM) supplemented with 10% fetal bovine serum (FBS) and 1% streptomycin/penicillin. The culture environment was set at 37°C, with 5% CO_2_. ADSCs from the third to fifth generation (P3-P5) were used in subsequent experiments. The ADSCs phenotype was confirmed by flow cytometry (Beckman Coulter, Fullerton, CA, United States ). Briefly, cells were washed with PBS, detached with trypsin, and incubated with phycoerythrin-conjugated CD29, CD90, CD105, CD34, CD45 and CD31. Isotype antibodies were applied as negative controls.

### Construction of Netrin-1-modified adipose-derived stem cells

Adenovirus purchased from Hanbio Biotechnology (Shanghai, China) was used to transfect Netrin-1 into ADSCs as previously described (Zhang et al., 2018). Briefly, HEK293 cells were used to generate the recombinant adenoviral vector green fluorescent protein (GFP)-Netrin-1 which was confirmed by RT-qPCR. GFP-Netrin-1 adenoviral vector was co-cultured with rat ADSCs at the multiplicity of infection (MOI) of 500 for 48 h. An inverted fluorescence microscope (Olympus IX81, Tokyo, Japan) was used to confirm the green fluorescence in transfected ADSCs, and the expression of Netrin-1 was detected by Western blotting and RT-qPCR, as previously described (Zhang et al., 2018).

### Viability assay of Netrin-1-modified adipose-derived stem cells cultured on adhesive double network hydrogel

To confirm the biosafety of our developed adhesive DN hydrogel, the proliferation and apoptosis of N-ADSCs were evaluated. Briefly, the adhesive DN hydrogel was seeded into a 96-well plate with 100 μl DMEM per well. The experimental group N-ADSCs (2 × 10^3^/well) was plated onto the adhesive DN hydrogel, while the control group consisted of the same number of N-ADSCs added in a well that did not contain the hydrogel. DMEM was changed every other day.

A cell counting kit-8 (CCK-8; Abcam, Cambridge, UK) was used to assess the cytotoxicity of the adhesive DN hydrogel. Briefly, 10 μl/well CCK-8 solution was added on days 1, 3 and 5 and incubated for 2 h at 37°C. The optical density (OD) at 450 nm wavelength was recorded using a microplate spectrophotometer (Varioskan; Thermo Fisher Scientific, Eugene, OR, United States).

For the apoptosis assay, N-ADSCs seeded with or without hydrogels were cultured for 48 h and the Annexin V-FITC/PI apoptosis detection kit (Abcam, Cambridge, United Kingdom) was used. Cells were analyzed using flow cytometry to detect the apoptosis ratio as previously mentioned (Zhang et al., 2018).

### Macrophage culture and polarization assay of macrophages co-cultured with Netrin-1-modified adipose-derived stem cells

The mouse macrophage cell line Raw264.7 was purchased from Sangon Biotech (Shanghai) Co., Ltd. and cultured in high-glucose DMEM supplemented with 10% FBS. Immunofluorescence and flow cytometry were used to detect the macrophage-specific antibody CD68 (Abcam, United Kingdom).

To assess the effect of ADSCs on macrophage polarization, Raw 264.7 cells (5 × 10^5^ cells) and N-ADSCs (10^5^ cells) were co-cultured in six-well transwell plates for 24 h as previously described (Yu et al., 2022). DMEM was used as a control. RT-qPCR was performed to examine the expression of Arginase-1 (Arg-1) and Interleukin 10 (IL-10) as polarization markers of M2 macrophages, while Interleukin 6 (IL-6), Tumor Necrosis Factor (TNF-α) were used as polarization markers of M1 macrophages. Then, 100 ng/ml lipopolysaccharide (LPS) was added for 4 h to stimulate Raw 264.7 cells—equal volumes of N-ADSC conditioned medium and DMEM were added into the wells. The expression of M2 macrophage marker CD206 (Abcam, United Kingdom) and M1 macrophage marker CD86 were quantified by flow cytometry and immunofluorescence to confirm the M1/M2 phenotype under a pro-inflammatory environment.

### Animal model and surgical procedures

The rat carotid artery guidewire injury model was established as described previously (Liang et al., 2019). Briefly, eighteen specific pathogen-free (SPF) male 10-week-old SD rats fed with a high-fat diet (21% fat, 0.15% cholesterol) were maintained at a constant temperature of 25°C for 1 week. First, rats were injected with 0.1 mg/100 g body weight of anticoagulant sodium heparin through the tail vein. Then, rats were anesthetized using 3% isoflurane and maintained under anesthesia using isoflurane plus 0.3% sodium pentobarbital (1 ml/100 g) i.p. The 18 rats were randomly divided into 3 groups: carotid artery wire injury control group, adhesive DN hydrogel group and N-ADSC + adhesive DN hydrogel group. For the control group, the skin was incised and the carotid artery was exposed and isolated. The left external carotid artery was incised, a 0.83 mm guidewire was inserted to the proximal end for about 1 cm, and the carotid endothelium was damaged by f5 gentle rotations of the wire. The vascular incision was closed with 8-0 sutures, the skin was closed with 3-0 silk sutures and sterilized with benzalkonium chloride. For the adhesive DN hydrogel group, a 1 × 1 cm adhesive DN hydrogel was wrapped around the damaged artery after the vascular incision was sutured, and 100 μl of DMEM was injected into the hydrogel with a syringe. For the N-ADSC + adhesive DN hydrogel group, 10^6^ N-ADSCs were diluted in 100 μl of DMEM and injected into the inner layer of hydrogel. After surgery animals were heated in an incubator and maintained a constant body temperature until recovery from anesthesia.

### Histological analysis

Rats were sacrificed 14 days after surgery, and the bilateral carotid arteries were isolated and processed for immunofluorescence staining. The integrity of the endothelium was analyzed by staining CD31 (Abcam, United Kingdom) and α-SMA (Abcam, United Kingdom). To further assess the distribution of M1 and M2 macrophages, macrophages were stained with F4/80 (Abcam, United Kingdom), M1 macrophages were stained with iNOS (Abcam, United Kingdom) and M2 macrophages were stained with CD206 (Abcam, United Kingdom). Nuclei were stained with 6-dimercapto-2-phenylindole (DAPI) (DAKO, USA). A Zeiss LSM 510 META immunofluorescence microscope (Carl-Zeiss-Strasse, Oberkochen, Germany) was used to observe and capture images. Carotid arteries were also paraffin-embedded and hematoxylin/eosin (HE) staining was performed. The ImageJ Pro Plus software (NIH, Bethesda, MD) was used to analyze the thickness of the carotid intima. In addition, the heart, liver, spleen, lung and kidney of rats were stained with HE to analyze the toxicity of the adhesive DN hydrogel to major organs.

### Statistical analysis

All quantitative data are presented as mean ± standard deviation (SD). Student’s *t*-test or one-way analysis of variance (ANOVA) was applied to compare differences between groups. *p* < 0.05 was defined as statistically significant. All experiments were repeated 3 times in each group.

## Results

### Morphology and physical properties of adhesive double network hydrogels

**FIGURE 1:**
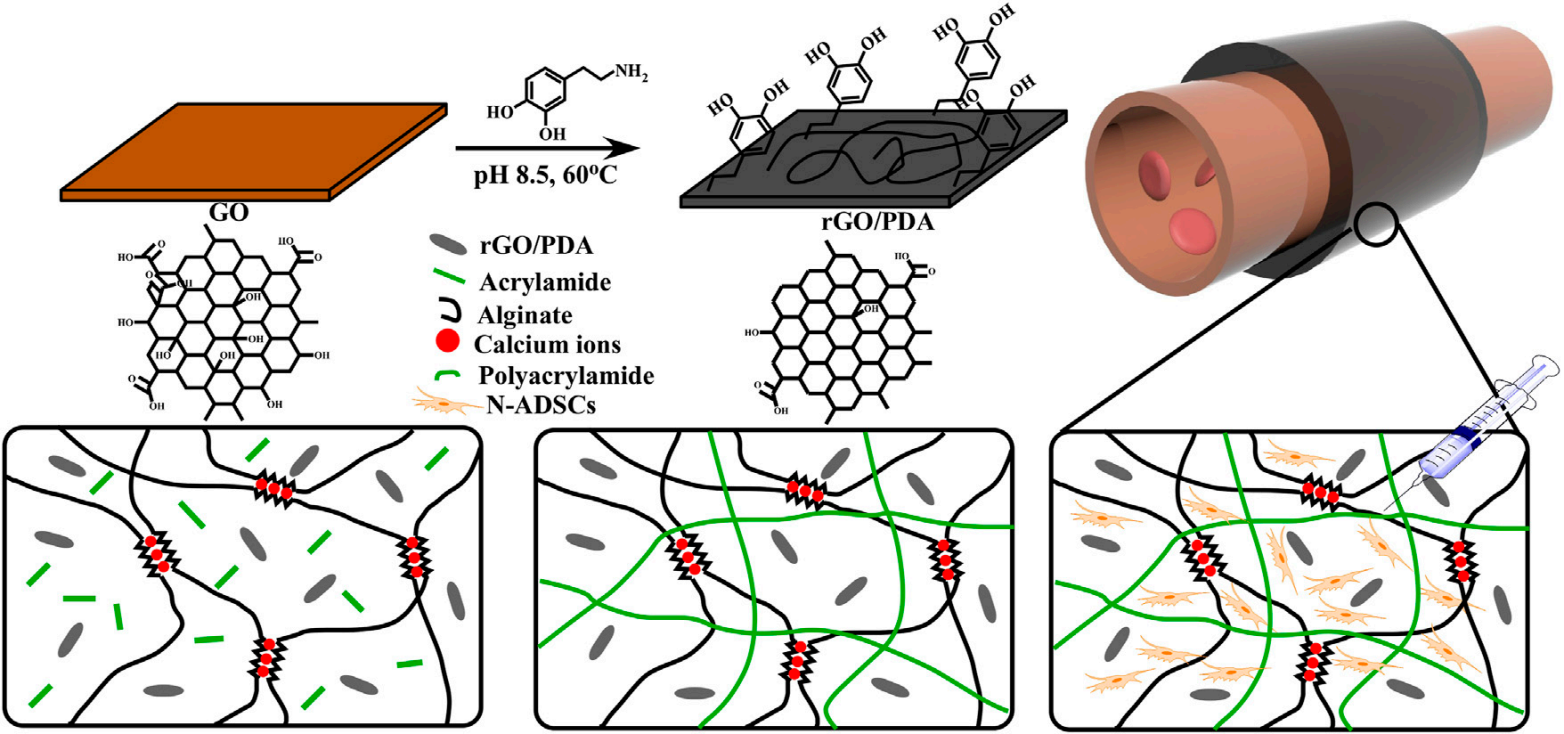
Schematics and fabrication process of the N-ADSC-loaded adhesive DN hydrogel. **(A)** Dopamine molecules were attached to graphene oxide (GO) sheets. **(B)** PDA chains were formed and GO was reduced. **(C)** The adhesive hydrogel was wrapped around an artery for repair. **(D,E)** Structure and polymerization process of the double network (DN) hydrogel. **(F)** N-ADSCs were injected into the hydrogel matrix. N-ADSCs, Netrin-1-modified adipose-derived stem cells.

**FIGURE 2:**
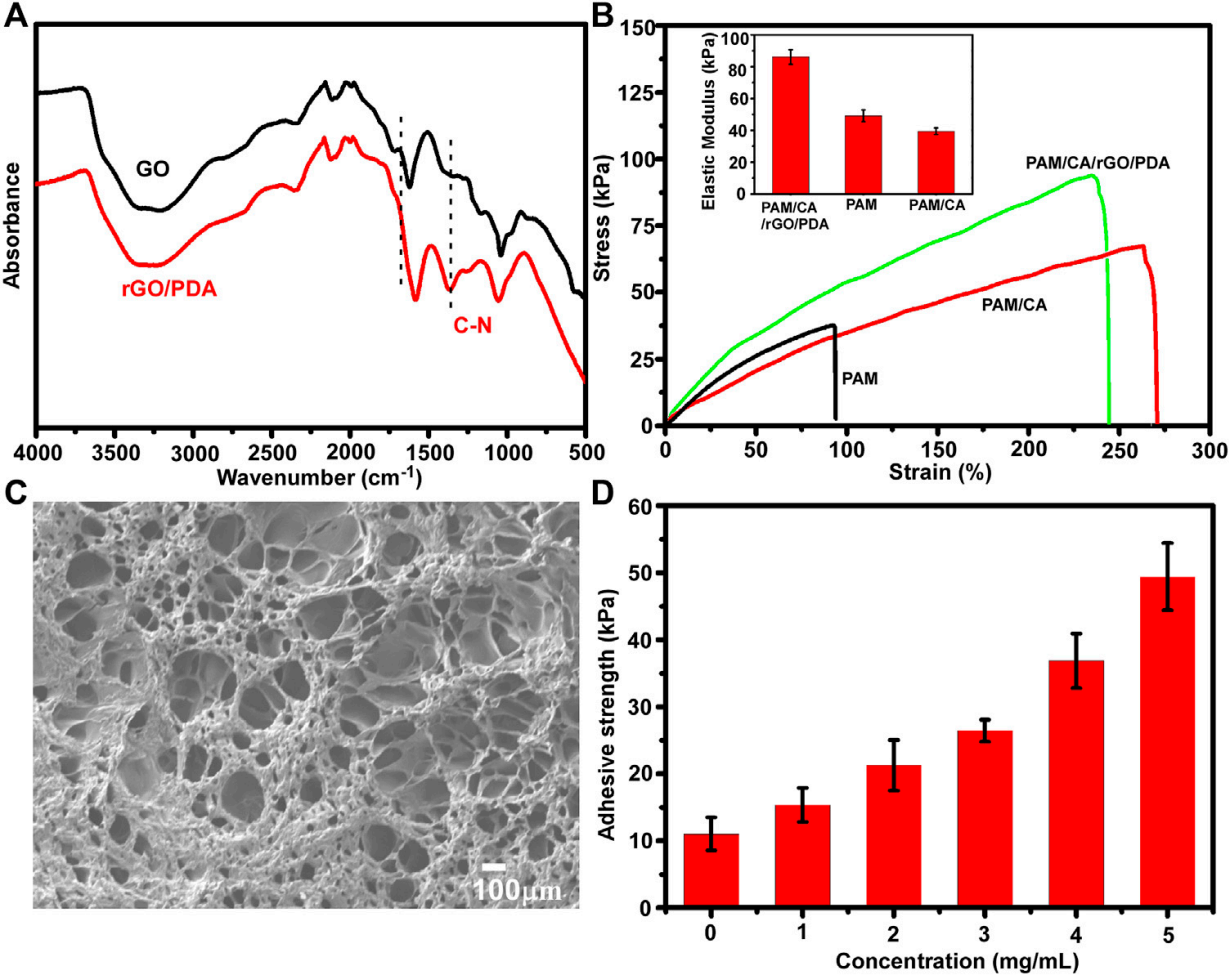
Characterization of the adhesive DN hydrogel. **(A)** FTIR spectra of dried GO and rGO/PDA. **(B)** Stress−strain curves of the DN hydrogel; insets show the elastic modulus. **(C)** SEM images of a dry adhesive DN hydrogel. **(D)** Adhesion strengths of the DN hydrogel onto porcine skin tissues with different concentrations of rGO/PDA.

The fabrication process of the adhesive DN hydrogel was schematically shown in Figure 1. Dopamine was intercalated and self-polymerized among GO sheets. The pace between GO sheets was limited, leading to a low volume of oxygen which hinders the overoxidation of PDA and maintains the massive free catechol groups to mimic the natural structures of mussels (Jin et al., 2021). FTIR was used to investigate the chemical structure of rGO/PDA (Figure 2A). Compared to GO, the peak intensity of rGO/PDA at 1,729 cm^−1^ was decreased which illustrates the reduction of GO by dopamine (Pandey et al., 2018). Furthermore, there was an obvious peak at 1,360 cm^−1^, which was affirmed as a C–N stretching vibration (Wan et al., 2017). Besides, rGO had fewer hydrophilic functional groups due to poor water solubility. As shown in Supplementary Figure S1, rGO/PDA was precipitated. Thus, the successful immobilization of PDA onto GO sheets by self-polymerization of dopamine was confirmed. The super mechanical performance of adhesive DN hydrogels was also investigated, as shown in Figure 2B. The ultimate tensile strength increased from 38.41 to 68.74 kPa, and the elongation at the break doubles enhanced from 91.82% to 269.32% in the DN hydrogel when compared to a single network (SN) PAM hydrogel. In addition, rGO/PDA proved to be an excellent reinforcing agent in the hydrogel matrix since it increased the elastic modulus from 39.31 ± 2.92 to 87.69 ± 2.92 kPa compared with the PAM/CA hydrogel. A similar elastic modulus to that of the arterial wall was shown to avoid local inflammatory responses caused by force mismatch at the interface between the scaffold and the tissue (Gulino et al., 2019).

Meanwhile, the morphology of the adhesive DN hydrogel was evaluated by SEM. As shown in Figure 2C, the adhesive DN hydrogel exhibited multiple porous structures which were vital for nutrient diffusion. The average pore size was around 113 ± 59 μm, with 70% porosity, which is suitable for cell retention and delivery (Eke et al., 2017). The adhesive capacity of the hydrogel was an important factor for long-term treatment and hemostasis. As depicted in Figure 2D, higher concentrations of rGO/PDA increased the adhesive strength of hydrogels, showing that hydrogel adhesiveness can be adjusted by varying the mass ratio of rGO/PDA. Compared with previous reports (Han et al., 2018; Hong et al., 2019), our hydrogel maintained an excellent adhesion to porcine skin (49.32 ± 5.48 kPa at 5 mg/ml) owing to the high binding affinity to diverse functional groups of peptides and proteins on the tissue surface (Ryu, et al., 2018). Altogether, the adhesive DN hydrogel could effectively bind to surrounding tissues and potentially maintain stem cell delivery *in vivo.*

### Characterization of adipose-derived stem cells and Netrin-1-modified adipose-derived stem cells

**FIGURE 3:**
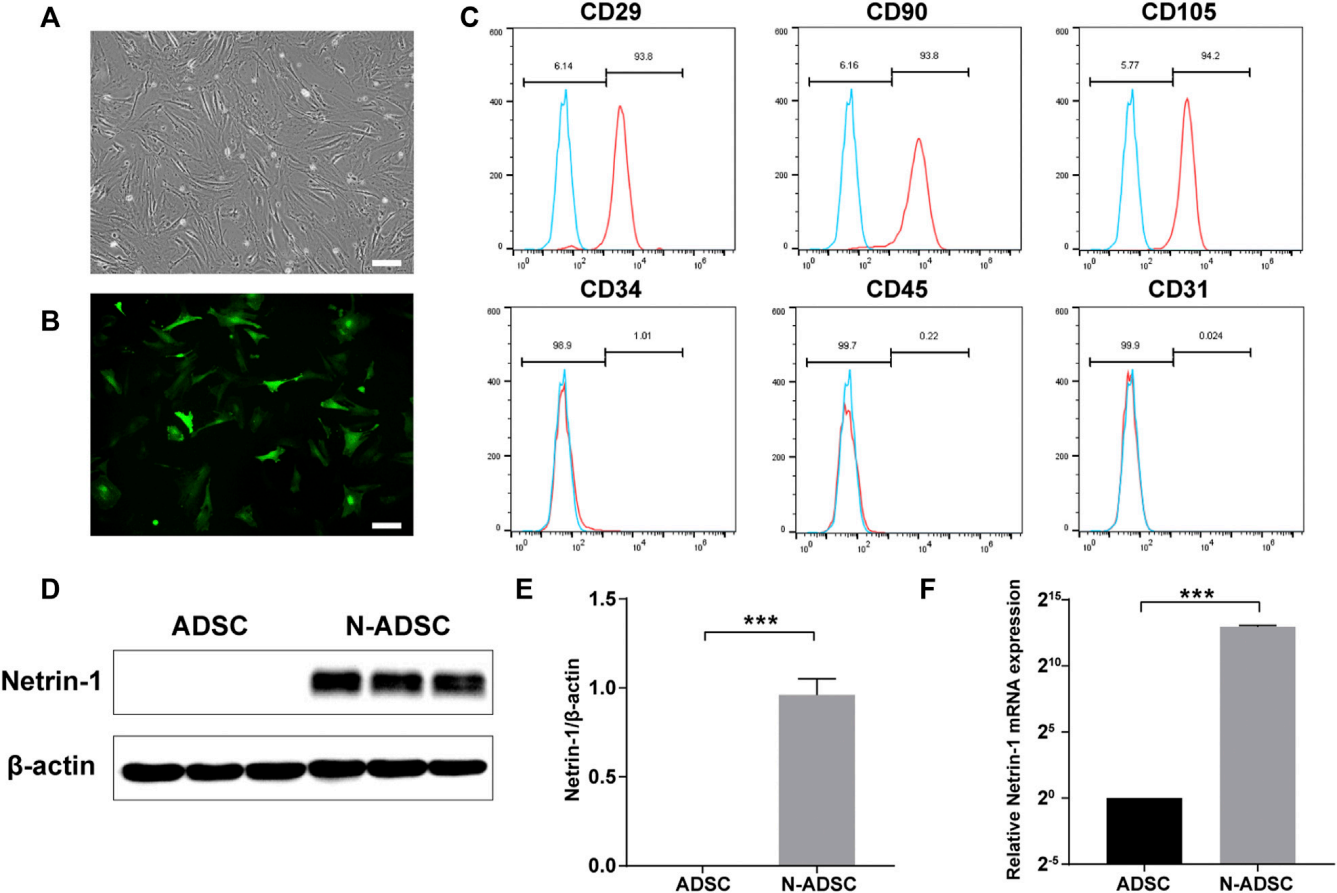
Characterization of ADSCs and N-ADSCs. **(A)** P3-ADSCs were examined under the microscope. **(B)** N-ADSCs were examined under a fluorescence microscope. **(C)** Identification of ADSC surface markers using flow cytometry. **(D,E)** Western blot and **(F)** RT-qPCR analyses proved the overexpression of Netrin-1 in the N-ADSCs. ***p < 0.001. Scale bar = 100 μm. P3, passage 3; N-ADSCs, Netrin-1-modified adipose-derived stem cells.

ADSCs were successfully isolated from inguinal and axillary adipose tissue of rats, and cultured until passages P3-P5. ADSCs demonstrated a typical spindle-like appearance (Figure 3A). Flow cytometric analysis showed that stem cell surface antigens CD29, CD90, and CD105 were positively expressed in ADSCs, while CD34, CD45, and the endothelial cell surface antigen CD31 were not expressed (Figure 3C). These are typical identification features of ADSCs.

The NTN-1 gene was successfully transfected into ADSCs using GFP-labeled adenovirus (Figure 3B). Adenoviruses have the advantages of low pathogenicity, no integration into the genome, high titer, and long gene expression duration (Zhang et al., 2018), which were ideal for our objectives. Western blotting and RT-qPCR were conducted and the results indicated that the expression of Netrin-1 (RNA and protein) was significantly upregulated in N-ADSCs compared with ADSCs (*P*< 0.001). It was remarkable that ADSCs showed a low expression of Netrin-1, thus corroborating our strategy of transfecting Netrin-1 into ADSCs (Figures 3D,E).

### The biocompatibility of adhesive double network hydrogels on Netrin-1-modified adipose-derived stem cells *in vitro*

**FIGURE 4:**
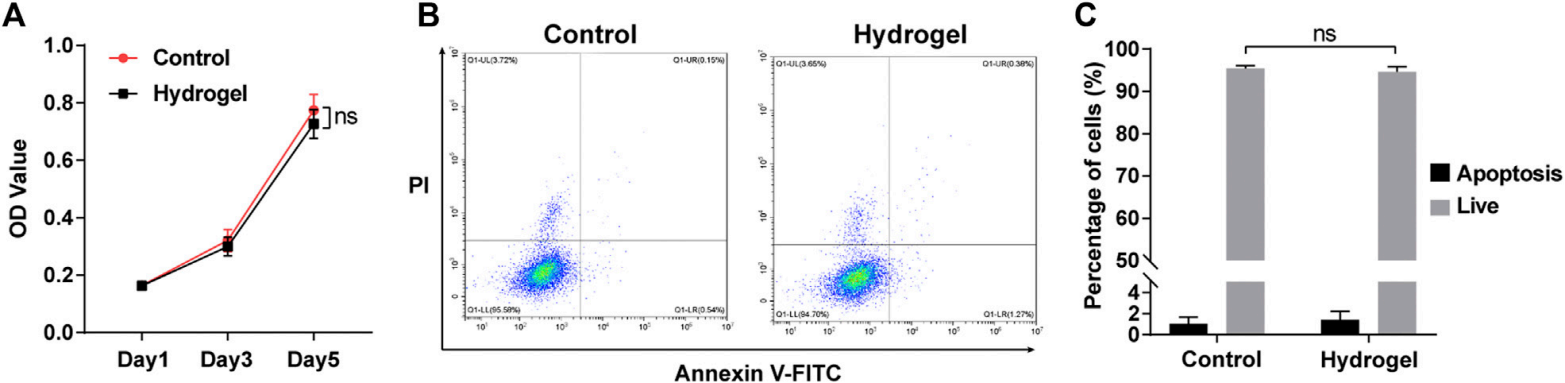
Biocompatibility of the adhesive DN hydrogel on N-ADSCs in vitro. **(A)** The CCK-8 proliferation assay of N-ADSCs seeded with or without the adhesive DN hydrogel. **(B,C)** Flowcytometry and statistical analysis of the apoptosis rate of N-ADSCs seeded with or without the adhesive DN hydrogel. ns, *p* > 0.05. N-ADSCs, Netrin-1-modified adipose-derived stem cells.

The CCK-8 proliferation assay showed that there was no significant difference between N-ADSCs seeded on wells coated with or without adhesive DN hydrogels for up to 5 days (*p* > 0.05) (Figure 4A). Flow cytometry analysis demonstrated that the apoptosis rate of N-ADSCs was also not affected when cells were seeded onto adhesive DN hydrogels (*p* > 0.05) (Figures 4B,C). Taken together, these assays corroborate that the adhesive DN hydrogel exhibited low cytotoxicity to N-ADSCs, which indicates satisfying biocompatibility.

### Characterization of macrophages and the effect of Netrin-1-modified adipose-derived stem cells on macrophage polarization *in vitro*

**FIGURE 5:**
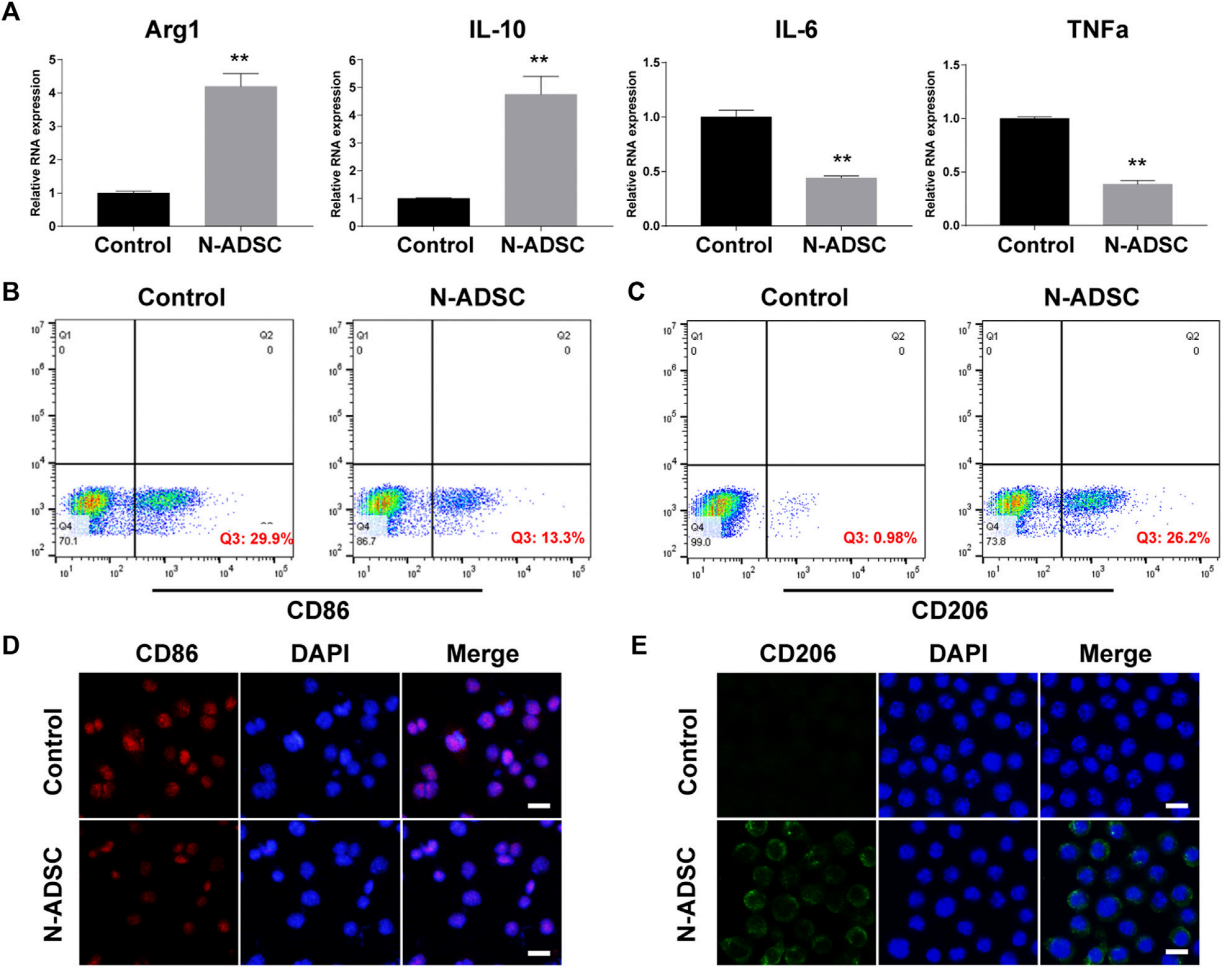
The effect of N-ADSCs on macrophage polarization in vitro. **(A)** RT-qPCR of Arg-1, IL-10, IL-6 and TNF-α from N-ADSCs-treated macrophages compared to DMEM-treated macrophages. **(B,C)** Addition of LPS and flow cytometry analysis of CD86 and CD206. **(D,E)** Immunofluorescence of CD86 and CD206. **p < 0.01. Scale bar = 100 μm. N-ADSCs, Netrin-1-modified adipose-derived stem cells.

Immunofluorescence detection and flow cytometry showed that Raw264.7 macrophages were strongly positive for the macrophage-specific antibody CD68, which validated the purity of these cells (Supplementary Figures S2A,B). The co-culture system of N-ADSCs and macrophages allowed N-ADSCs to influence macrophages via a paracrine effect, which was reported to be the main effect of ADSCs after transplantation *in vivo* (Cai et al., 2020). After being co-cultured for 24 h, M2 markers Arg-1 and IL-10 were significantly up-regulated while M1 markers IL-6 and TNF-α were significantly downregulated in N-ADSCs-treated macrophages compared to DMEM-treated ones (*p* < 0.05) (Figure 5A). This indicates that under normal circumstances N-ADSCs significantly induce the M2 polarization of macrophages to decrease inflammation. Considering the pro-inflammatory microenvironment of carotid intima injury, LPS was added into the culture medium to mimic arterial inflammation, and results of flow cytometry (Figures 5B,C) and immunofluorescence (Figures 5D,E) experiments demonstrated that CD206 was significantly upregulated and CD86 was downregulated. This depicts an increase in M2 macrophages due to an M2 polarization or an M1-to-M2 phenotype transition induced by N-ADSCs.

### The effect of N-ADSC-loaded adhesive double network hydrogels on macrophage polarization and intimal hyperplasia *in vivo*

**FIGURE 6:**
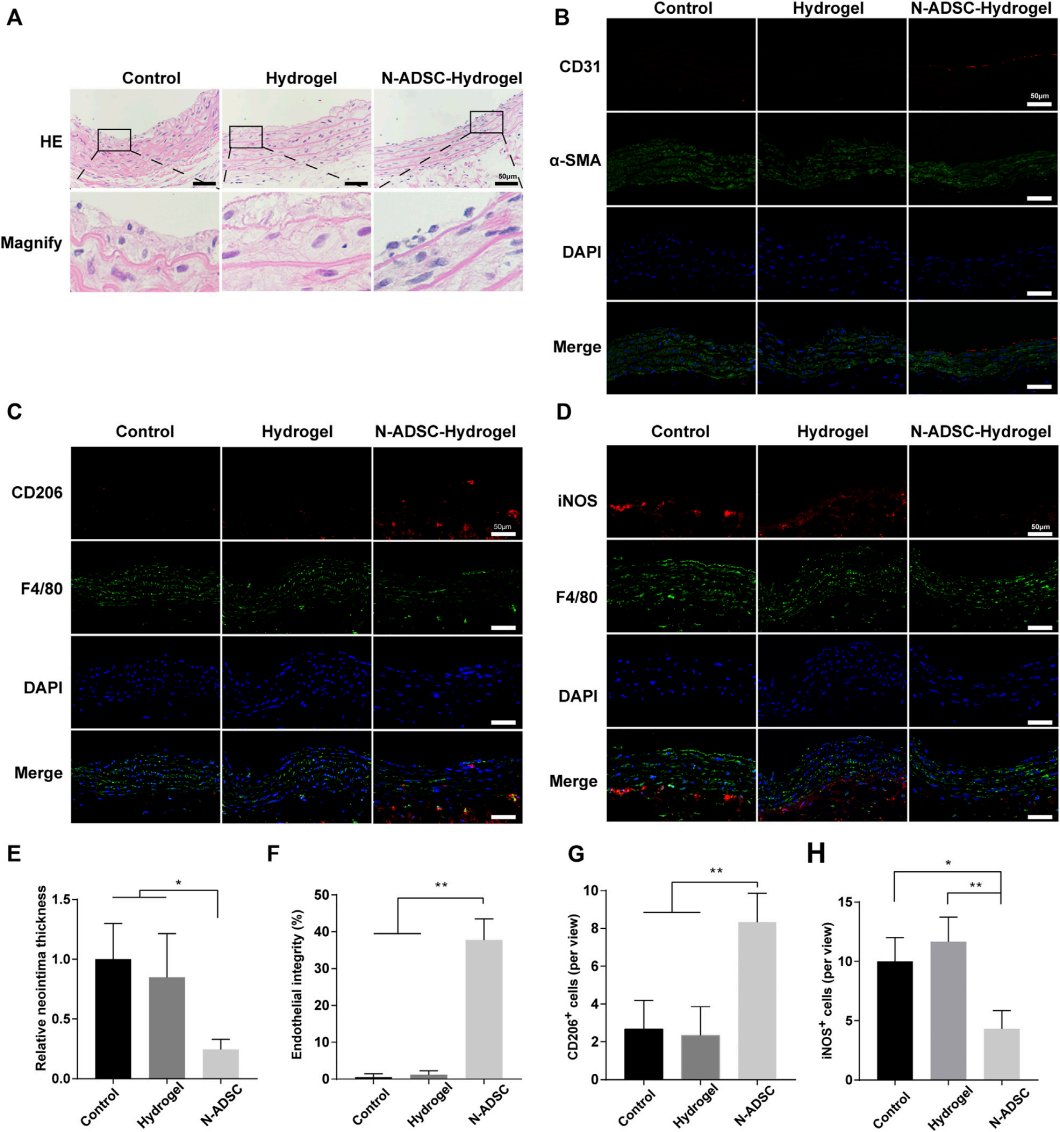
The effect of N-ADSC-loaded adhesive DN hydrogels on macrophage polarization and intimal hyperplasia *in vivo*. **(A,E)** HE staining and statistical analysis of the thickness of the carotid neointima. **(B,F)** Immunofluorescence staining of CD31 (red) and α-SMA (green) depicting endothelial integrity. **(C, G)** Immunofluorescence staining of CD206 (red) and F4/80 (green) and analysis of CD206+ cells. **(D,H)** Immunofluorescence staining of iNOS (red) and F4/80 (green) and analysis of iNOS + cells. *p < 0.05. **p < 0.01. Scale bar = 50 μm. N-ADSCs, Netrin-1-modified adipose-derived stem cells.

The rat carotid artery guidewire/balloon injury model is acknowledged to mimic the carotid interventional injury that occurs in patients. Therefore, a guidewire was applied due to its small diameter that facilitates handling. Indeed, the thickness of both tunica neointima and tunica media was significantly reduced in N-ADSCs + adhesive DN hydrogel rats when compared to other groups. Moreover, the texture of the tunica media was smoother and more continuous in N-ADSCs + adhesive DN hydrogel rats (Figures 6A,E; Supplementary Figure S4), which indicates less damage to vascular endothelial cells, a lower proliferation of vascular smooth muscle cells and less macrophage infiltration. Besides, the integrity of the endothelium was partially restored in the N-ADSCs + adhesive DN hydrogel group when compared to that of the other two groups (Figures 6B,F). Immunofluorescence staining of CD206, iNOS, and F4/80 evidenced that N-ADSCs + adhesive DN hydrogel rats had more M2 and less M1 macrophages than the other two groups (Figures 6C,D,G,H). This suggests that a combination of N-ADSCs plus the adhesive DN hydrogel promoted the M2 polarization and inhibited the M1 polarization of macrophages, thus inhibiting inflammation. Taken together, the above results corroborated that the combination of N-ADSCs and the adhesive DN hydrogel reduced arterial inflammation and inhibited intimal hyperplasia after endovascular interventions *in vivo*.

### The biocompatibility of N-ADSC-loaded adhesive double network hydrogels *in vivo*

**FIGURE 7:**
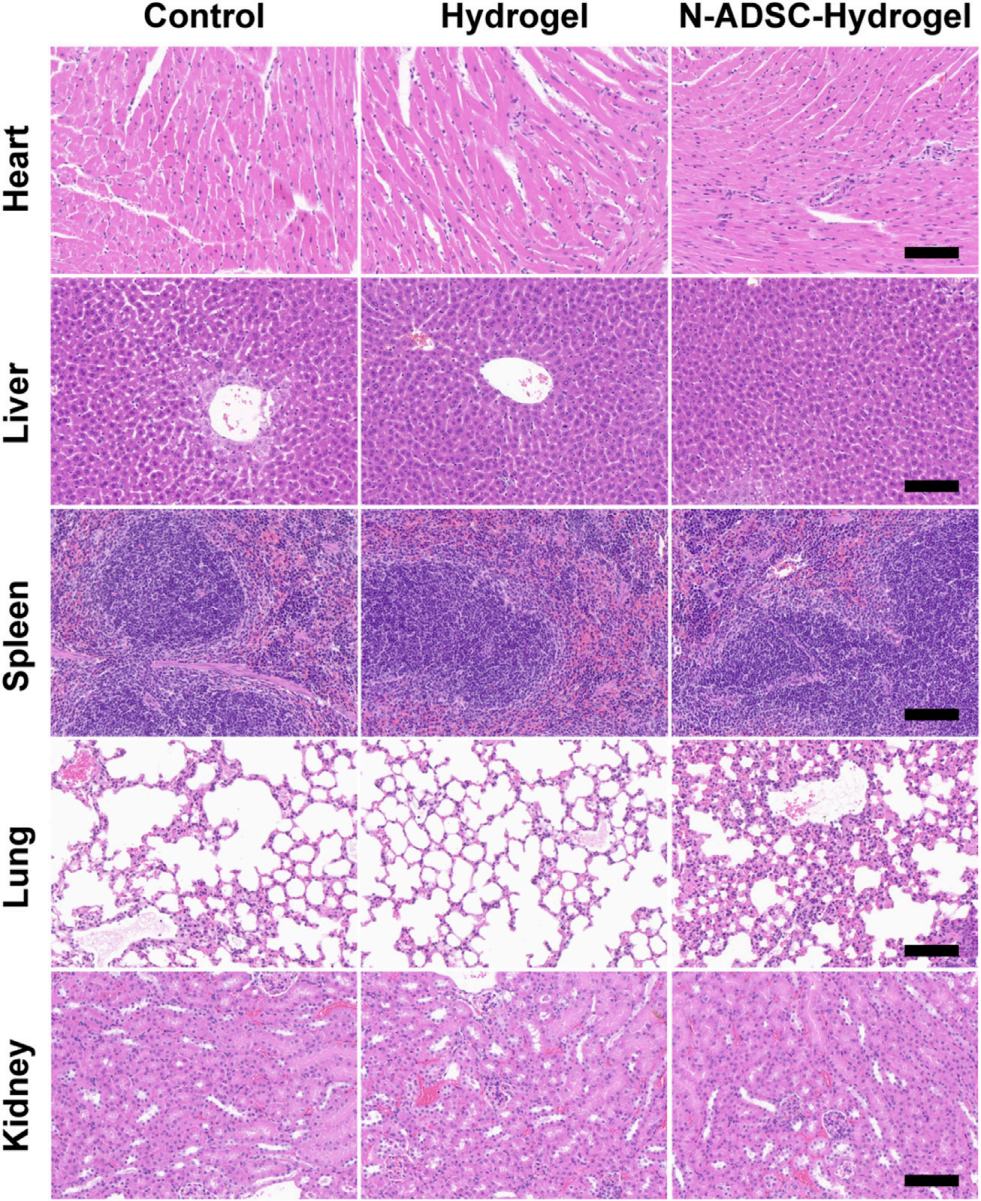
Biocompatibility of the N-ADSC-loaded adhesive DN hydrogel *in vivo*. HE staining of the heart, liver, spleen, lung and kidney from rats of the three experimental groups as detailed in Materials and Methods.

Excellent biocompatibility is essential for a safe and qualified implantation material. To examine the *in vivo* biocompatibility of the N-ADSC-loaded adhesive DN hydrogel, major organs were collected and HE stained from rats subjected to surgery and treated with or without the N-ADSC-loaded adhesive DN hydrogel. There were no significant differences in the heart, liver, spleen, lung and kidney in terms of lesions, injuries or inflammation among groups (Figure 7). This testifies that the N-ADSC-loaded adhesive DN hydrogel had no biotoxicity and showed satisfying biocompatibility *in vivo*.

## Discussion

We report a biocompatible slow-releasing system of N-ADSC-loaded adhesive DN hydrogel wrap which significantly promoted the M2 polarization of macrophages, reduced arterial inflammation, inhibited intimal and media hyperplasia, and improved the re-endothelialization in a rat carotid intima injury model. Results of the present study provide a new strategy to treat arterial restenosis after endovascular interventions in clinical practice.

We designed a PAM/CA/rGO/PDA adhesive DN hydrogel that shows several advantages as a platform to load N-ADSCs. The rGO/PDA was inspired by the mussel adhesive mechanism and showed a high adhesiveness to tissue surfaces thus allowing the hydrogel to tightly wrap around damaged arteries for long-term treatment**.** Besides, rGO had three key functions in hydrogels: it prevented PDA overoxidation, enhanced the stiffness of the hydrogel and promoted the adhesion and growth of stem cells (Lee et al., 2011). The DN design of a PAM/CA hydrogel was endowed with suitable toughness and stretch ability because of effective energy dissipation (Nakayama et al., 2004). Meanwhile, the abundant porous structure of the DN hydrogel improved stem cell retention and sustainable delivery.

We obtained stable N-ADSCs overexpressing Netrin-1. Our previously published study (Zhang et al., 2018) found that Netrin-1 improved ADSCs proliferation, migration, adhesion, and inhibited apoptosis through the activation of the AKT/PI3K/eNOS signaling pathway, and secretion of VEGF, b-FGF, HGF, PDGF, EGF and IGF-1. Therefore, the overexpression of Netrin-1 improved the biological functions of ADSCs. At the same time, our present report shows that N-ADSCs expressed a large amount of Netrin-1, while the secreted Netrin-1 protein was shown to induce M2 macrophage polarization (Li et al., 2017) and attenuate the neointimal formation (Liu et al., 2017). Thus, N-ADSCs are key to induce the M2 polarization of macrophages and secrete anti-inflammation cytokines.

Currently, intensive research output has focused on perivascular drug delivery systems. Several approaches, such as targeted drugs, films/wraps, depot gels, meshes, rings, or micro/nanoparticles have been proposed. Many of these have shown promising efficacy and safety to alleviate intimal hyperplasia through the perivascular delivery of drugs (Lee et al., 2017). Indeed, Christopher et al. used a micro-infusion catheter to inject adventitial drug into the vasculature and found that this approach was safe and feasible for adjunctive therapy in the femoropopliteal artery (Owens et al., 2014). Meanwhile, William et al. constructed a 30-day reabsorbable polymer-based bilayer wrap loaded with sunitinib for perivascular drug delivery (Sanders et al., 2012). In a rabbit carotid artery bypass model, Wu et al. (2018) constructed a resolvin D1-loaded perivascular Pluronic F127 gel to attenuate venous graft hyperplasia without biotoxicity. Warren et al. found that the injection of NAB-rapamycin into the tunica adventitia vasorum could inhibit medial proliferation and adventitial inflammation after balloon angioplasty in porcine arteries to reduce lumen stenosis (Gasper et al., 2013). However, these previous studies were focused on the perivascular delivery of antiproliferative drugs, which may be toxic to blood vessels and have a short drug half-life. Here we used N-ADSCs as a “drug.” Compared with pure Netrin-1 protein or other anti-proliferative drugs, N-ADSCs provide a “biological pump” that stably secrete Netrin-1 and other anti-inflammatory cytokines in large quantities. In parallel, the adhesive DN hydrogel served as an excellent three-dimensional scaffold and natural culture medium for N-ADSCs with strong adhesion to the surface of blood vessels, and controlled release and distribution of Netrin-1 and other cytokines into the vascular wall. These strategies synergized to create a slow-release mechanism to reduce intimal hyperplasia.

The premise of evaluating the effectiveness of a biomaterial is to first assess its biosafety. The biocompatibility and biodegradability of graphene-based materials are still controversial. Pinto et al. (2013) found a slight decrease in cell viability in bacteria and mammalian cells after exposure to graphene-based materials, while Park et al. (2014) found that graphene promoted mesenchymal stem cells differentiation into cardiomyocytes by enhancing the expression of extracellular matrix proteins. Previous studies have also shown that peroxidase secreted by activated immune cells such as neutrophils and eosinophils *in vivo* can biodegrade graphene (Kurapati et al., 2018). Therefore, we carefully evaluated the biocompatibility and toxicity of the adhesive DN hydrogel applied in this study. We showed no cytotoxicity *in vitro* as well as no deleterious effect on major organs *in vivo* upon the use of our developed DN hydrogel. Therefore, our adhesive DN hydrogel is a material with excellent biocompatibility and biodegradation.

## Conclusion

In conclusion, the results of this study corroborate that the adhesive DN hydrogel wrap loaded with N-ADSCs can significantly reduce arterial inflammation, inhibit intimal hyperplasia and improve re-endothelialization. Hence, the slow-releasing system of N-ADSCs-loaded adhesive DN hydrogel wrap was safe and effective, thus being characterized as a promising and novel therapeutic approach to treat vascular restenosis after endovascular interventions.

## Data Availability

The raw data supporting the conclusion of this article will be made available by the authors, without undue reservation.

## Supplementary material

Supplementary Figure S1 Difference in water solubility of GO and rGO/PDA.

Supplementary Figure S2 Characterization of Raw264.7 cells. **(A)** Immunofluorescence of CD68. **(B)** Flow cytometry of CD68.

Supplementary Figure S3 Surgical operation and development of the rat model. **(A)** The N-ADSC-loaded adhesive DN hydrogel was successfully wrapped around the carotid artery of rats. **(B)** Rats were sacrificed 14 days after operation and bilateral carotid arteries were isolated.

Supplementary Figure S4 Relative media thickness, a supplement to Figure 6A. **p* < 0.05.

Supplementary Figure S5 Immunofluorescence staining of CD31 (red) and α-SMA (green) depicting endothelial integrity of control group (no injury and no treatment).

## References

[B1] Adamiak M. Cheng G. Bobis-Wozowicz S. Zhao L. Kedracka-Krok S. Samanta A. (2018). Induced pluripotent stem cell (iPSC)-Derived extracellular vesicles are safer and more effective for cardiac repair than iPSCs. Circ. Res. 122 (2), 296–309. 10.1161/CIRCRESAHA.117.311769 29118058 PMC5775034

[B2] Bhattacharjee M. Escobar Ivirico J. L. Kan H. M. Shah S. Otsuka T. Bordett R. (2022). Injectable amnion hydrogel-mediated delivery of adipose-derived stem cells for osteoarthritis treatment. Proc. Natl. Acad. Sci. U. S. A. 119 (4), e2120968119. 10.1073/pnas.2120968119 35046053 PMC8794776

[B3] Cai Y. Li J. Jia C. He Y. Deng C. (2020). Therapeutic applications of adipose cell-free derivatives: a review. Stem Cell Res. Ther. 11 (1), 312. 10.1186/s13287-020-01831-3 32698868 PMC7374967

[B4] Conte M. S. Bradbury A. W. Kolh P. White J. V. Dick F. Fitridge R. (2019). Global vascular Guidelines on the management of chronic limb-threatening ischemia. Eur. J. Vasc. Endovasc. Surg. 58 (1S), S1–S109. 10.1016/j.ejvs.2019.05.006 31182334 PMC8369495

[B5] Ding Q. Liao S. J. Yu J. (2014). Axon guidance factor netrin-1 and its receptors regulate angiogenesis after cerebral ischemia. Neurosci. Bull. 30 (4), 683–691. 10.1007/s12264-013-1441-9 24875332 PMC5562620

[B6] Eke G. Mangir N. Hasirci N. MacNeil S. Hasirci V. (2017). Development of a UV crosslinked biodegradable hydrogel containing adipose derived stem cells to promote vascularization for skin wounds and tissue engineering. Biomaterials 129, 188–198. 10.1016/j.biomaterials.2017.03.021 28343005

[B7] Gasper W. J. Jimenez C. A. Walker J. Conte M. S. Seward K. Owens C. D. (2013). Adventitial nab-rapamycin injection reduces porcine femoral artery luminal stenosis induced by balloon angioplasty via inhibition of medial proliferation and adventitial inflammation. Circ. Cardiovasc. Interv. 6 (6), 701–709. 10.1161/CIRCINTERVENTIONS.113.000195 24221390 PMC3888086

[B8] Ghandforoushan P. Golafshan N. Babu Kadumudi F. Castilho M. Dolatshahi-Pirouz A. Orive G. (2022). Injectable and adhesive hydrogels for dealing with wounds. Expert Opin. Biol. Ther. 22 (4), 519–533. 10.1080/14712598.2022.2008353 34793282

[B9] Gnecchi M. Danieli P. Malpasso G. Ciuffreda M. C. (2016). Paracrine mechanisms of mesenchymal stem cells in tissue repair. Methods Mol. Biol. 1416, 123–146. 10.1007/978-1-4939-3584-0_7 27236669

[B10] Gu Z. Huang K. Luo Y. Zhang L. Kuang T. Chen Z. (2018). Double network hydrogel for tissue engineering. Wiley Interdiscip. Rev. Nanomed. Nanobiotechnol. 10 (6), e1520. 10.1002/wnan.1520 29664220

[B11] Gulino M. Kim D. Pané S. Santos S. D. Pêgo A. P. (2019). Tissue response to neural implants: the use of model systems toward new design solutions of implantable microelectrodes. Front. Neurosci. 13, 689. 10.3389/fnins.2019.00689 31333407 PMC6624471

[B12] Han L. Lu X. Liu K. Wang K. Fang L. Weng L. T. (2017). Mussel-inspired adhesive and tough hydrogel based on nanoclay confined dopamine polymerization. ACS Nano 11 (3), 2561–2574. 10.1021/acsnano.6b05318 28245107

[B13] Han L. Wang M. Li P. Gan D. Yan L. Xu J. (2018). Mussel-inspired tissue-adhesive hydrogel based on the polydopamine-chondroitin sulfate complex for growth-factor-free cartilage regeneration. ACS Appl. Mat. Interfaces 10 (33), 28015–28026. 10.1021/acsami.8b05314 30052419

[B14] He X. Dong Z. Cao Y. Wang H. Liu S. Liao L. (2019). MSC-derived exosome promotes M2 polarization and enhances cutaneous wound healing. Stem Cells Int. 2019, 1–16. 10.1155/2019/7132708 PMC6754952 31582986

[B15] Hong Y. Zhou F. Hua Y. Zhang X. Ni C. Pan D. (2019). A strongly adhesive hemostatic hydrogel for the repair of arterial and heart bleeds. Nat. Commun. 10 (1), 2060. 10.1038/s41467-019-10004-7 31089131 PMC6517429

[B16] Jin X. Jiang H. Zhang Z. Yao Y. Bao X. Hu Q. (2021). Ultrastretchable, self-adhesive, strain-sensitive and self-healing GO@DA/Alginate/P(AAc-co-AAm) multifunctional hydrogels via mussel-inspired chemistry. Carbohydr. Polym. 254, 117316. 10.1016/j.carbpol.2020.117316 33357879

[B17] Kamann S. Haase T. Stolzenburg N. Löchel M. Peters D. Schnorr J. (2019). Resveratrol-coated balloon catheters in porcine coronary and peripheral arteries. Int. J. Mol. Sci. 20 (9), 2285. 10.3390/ijms20092285 PMC6540025 31075824

[B18] Krankenberg H. Tübler T. Ingwersen M. Schlüter M. Scheinert D. Blessing E. (2015). Drug-coated balloon versus standard balloon for superficial femoral artery in-stent restenosis: the randomized femoral artery in-stent restenosis (FAIR) trial. Circulation 132 (23), 2230–2236. 10.1161/CIRCULATIONAHA.115.017364 26446728

[B19] Kurapati R. Mukherjee S. P. Martín C. Bepete G. Vázquez E. Pénicaud A. (2018). Degradation of single-layer and few-layer graphene by neutrophil myeloperoxidase. Angew. Chem. Int. Ed. 57 (36), 11722–11727. 10.1002/anie.201806906 30006967

[B20] Lee W. C. Lim C. H. Shi H. Tang L. A. Wang Y. Lim C. T. (2011). Origin of enhanced stem cell growth and differentiation on graphene and graphene oxide. ACS Nano 5 (9), 7334–7341. 10.1021/nn202190c 21793541

[B21] Lee J. Kim D. H. Lee K. J. Seo I. H. Park S. H. Jang E. H. (2017). Transfer-molded wrappable microneedle meshes for perivascular drug delivery. J. Control. Release 268, 237–246. 10.1016/j.jconrel.2017.10.007 29030224

[B22] Li Y. Wan S. Liu G. Cai W. Huo D. Li G. (2017). Netrin-1 promotes inflammation resolution to achieve endothelialization of small-diameter tissue engineering blood vessels by improving endothelial progenitor cells function *in situ* . Adv. Sci. (Weinh) 4 (12), 1700278. 10.1002/advs.201700278 29270340 PMC5738088

[B23] Liang J. Li Q. Cai W. Zhang X. Yang B. Li X. (2019). Inhibition of polycomb repressor complex 2 ameliorates neointimal hyperplasia by suppressing trimethylation of H3K27 in vascular smooth muscle cells. Br. J. Pharmacol. 176 (17), 3206–3219. 10.1111/bph.14754 31162630 PMC6692584

[B24] Liu N. M. Siu K. L. Youn J. Y. Cai H. (2017). Attenuation of neointimal formation with netrin-1 and netrin-1 preconditioned endothelial progenitor cells. J. Mol. Med. 95 (3), 335–348. 10.1007/s00109-016-1490-4 28004124 PMC5976243

[B25] Nakayama A. Kakugo A. Gong J. P. Osada Y. Takai M. Erata T. (2004). High mechanical strength double network hydrogel with bacterial cellulose. Adv. Funct. Mat. 14, 1124–1128. 10.1002/adfm.200305197

[B26] Owens C. D. Gasper W. J. Walker J. P. Alley H. F. Conte M. S. Grenon S. M. (2014). Safety and feasibility of adjunctive dexamethasone infusion into the adventitia of the femoropopliteal artery following endovascular revascularization. J. Vasc. Surg. 59 (4), 1016–1024. 10.1016/j.jvs.2013.10.051 24423476 PMC4391811

[B27] Pandey N. Hakamivala A. Xu C. Hariharan P. Radionov B. Huang Z. (2018). Biodegradable nanoparticles enhanced adhesiveness of mussel-like hydrogels at tissue interface. Adv. Healthc. Mat. 7 (7), e1701069. 10.1002/adhm.201701069 PMC5902656 29205950

[B28] Park J. Park S. Ryu S. Bhang S. H. Kim J. Yoon J. K. (2014). Graphene-regulated cardiomyogenic differentiation process of mesenchymal stem cells by enhancing the expression of extracellular matrix proteins and cell signaling molecules. Adv. Healthc. Mat. 3 (2), 176–181. 10.1002/adhm.201300177 23949999

[B29] Pinto A. M. Gonçalves I. C. Magalhães F. D. (2013). Graphene-based materials biocompatibility: a review. Colloids Surfaces B Biointerfaces 111, 188–202. 10.1016/j.colsurfb.2013.05.022 23810824

[B30] Ranganathan P. V. Jayakumar and C. Ramesh G. (2013). Netrin-1-treated macrophages protect the kidney against ischemia-reperfusion injury and suppress inflammation by inducing M2 polarization. Am. J. Physiol. Renal Physiol. 304 (7), F948–F957. 10.1152/ajprenal.00580.2012 23408164 PMC3625850

[B31] Razavi M. K. Donohoe D. D'Agostino R. B. Jaff M. R. Adams G. DANCE Investigators (2018). Adventitial drug delivery of dexamethasone to improve primary patency in the treatment of superficial femoral and popliteal artery disease: 12-Month results from the DANCE clinical trial. JACC Cardiovasc. Interv. 11 (10), 921–931. 10.1016/j.jcin.2017.12.015 29730377

[B32] Ryu J. H. Messersmith P. B. Lee H. (2018). Polydopamine surface chemistry: a decade of discovery. ACS Appl. Mat. Interfaces 10 (9), 7523–7540. 10.1021/acsami.7b19865 PMC6320233 29465221

[B33] Sanders W. G. Hogrebe P. C. Grainger D. W. Cheung A. K. Terry C. M. (2012). A biodegradable perivascular wrap for controlled, local and directed drug delivery. J. Control. Release 161 (1), 81–89. 10.1016/j.jconrel.2012.04.029 22561340 PMC3378780

[B34] Sinha S. K. Miikeda A. Fouladian Z. Mehrabian M. Edillor C. Shih D. (2021). Local M-CSF (macrophage colony-stimulating factor) expression regulates macrophage proliferation and apoptosis in atherosclerosis. Arterioscler. Thromb. Vasc. Biol. 41 (1), 220–233. 10.1161/ATVBAHA.120.315255 33086870 PMC7769919

[B35] Tabas I. Lichtman A. H. (2017). Monocyte-Macrophages and T Cells in atherosclerosis. Immunity 47 (4), 621–634. 10.1016/j.immuni.2017.09.008 29045897 PMC5747297

[B36] Vo T. N. Kasper F. K. Mikos A. G. (2012). Strategies for controlled delivery of growth factors and cells for bone regeneration. Adv. Drug Deliv. Rev. 64 (12), 1292–1309. 10.1016/j.addr.2012.01.016 22342771 PMC3358582

[B37] Wan Q. Liu M. Xie Y. Tian J. Huang Q. Deng F. (2017). Facile and highly efficient fabrication of graphene oxide-based polymer nanocomposites through mussel-inspired chemistry and their environmental pollutant removal application. J. Mat. Sci. 52, 504–518. 10.1007/s10853-016-0349-y

[B38] Wu B. Werlin E. C. Chen M. Mottola G. Chatterjee A. Lance K. D. (2018). Perivascular delivery of resolvin D1 inhibits neointimal hyperplasia in a rabbit vein graft model. J. Vasc. Surg. 68 (6S), 188S–200S. 10.1016/j.jvs.2018.05.206 30064835 PMC6252159

[B39] Xia X. Hu Z. Wang S. Yin K. (2022). Netrin-1: an emerging player in inflammatory diseases. Cytokine Growth Factor Rev. 64, 46. 10.1016/j.cytogfr.2022.01.003 35082104

[B40] Yahagi K. Otsuka F. Sakakura K. Sanchez O. D. Kutys R. Ladich E. (2014). Pathophysiology of superficial femoral artery in-stent restenosis. J. Cardiovasc. Surg. 55 (3), 307–323. 10.1016/j.cardfail.2014.04.015 24755699

[B41] Yan W. Li T. Yin T. Hou Z. Qu K. Wang N. (2020). M2 macrophage-derived exosomes promote the c-KIT phenotype of vascular smooth muscle cells during vascular tissue repair after intravascular stent implantation. Theranostics 10 (23), 10712–10728. 10.7150/thno.46143 32929376 PMC7482821

[B42] Yu L. Shi Q. Zhang B. Xu J. (2022). Genetically modified mesenchymal stem cells promote spinal fusion through polarized macrophages. Lab. Invest. 102 (3), 312–319. 10.1038/s41374-021-00693-4 34764437 PMC8860744

[B43] Zhang X. Qin J. Wang X. Guo X. Liu J. Wang X. (2018). Netrin-1 improves adipose-derived stem cell proliferation, migration, and treatment effect in type 2 diabetic mice with sciatic denervation. Stem Cell Res. Ther. 9 (1), 285. 10.1186/s13287-018-1020-0 30359296 PMC6202825

[B44] Zhang X. Liu J. Yang X. He G. Li B. Qin J. (2019). CuCo_2_S_4_ nanocrystals as a nanoplatform for photothermal therapy of arterial inflammation. Nanoscale 11 (19), 9733–9742. 10.1039/c9nr00772e 31066405

[B45] Zhu J. Marchant R. E. (2011). Design properties of hydrogel tissue-engineering scaffolds. Expert Rev. Med. Devices 8 (5), 607–626. 10.1586/erd.11.27 22026626 PMC3206299

[B46] Zhuang M. Ou X. Dou Y. Zhang L. Zhang Q. Wu R. (2016). Polymer-embedded fabrication of Co2P nanoparticles encapsulated in N, P-doped graphene for hydrogen generation. Nano Lett. 16 (7), 4691–4698. 10.1021/acs.nanolett.6b02203 27267432

