# Supplementary figures and images for: Adhesive hydrogel wrap loaded with Netrin-1-modified adipose-derived stem cells: An effective approach against periarterial inflammation after endovascular intervention
Source: Front Bioeng Biotechnol. 2022 Jul 22;10:944435. doi: 10.3389/fbioe.2022.944435 (PMC9355160; doi:10.3389/fbioe.2022.944435)

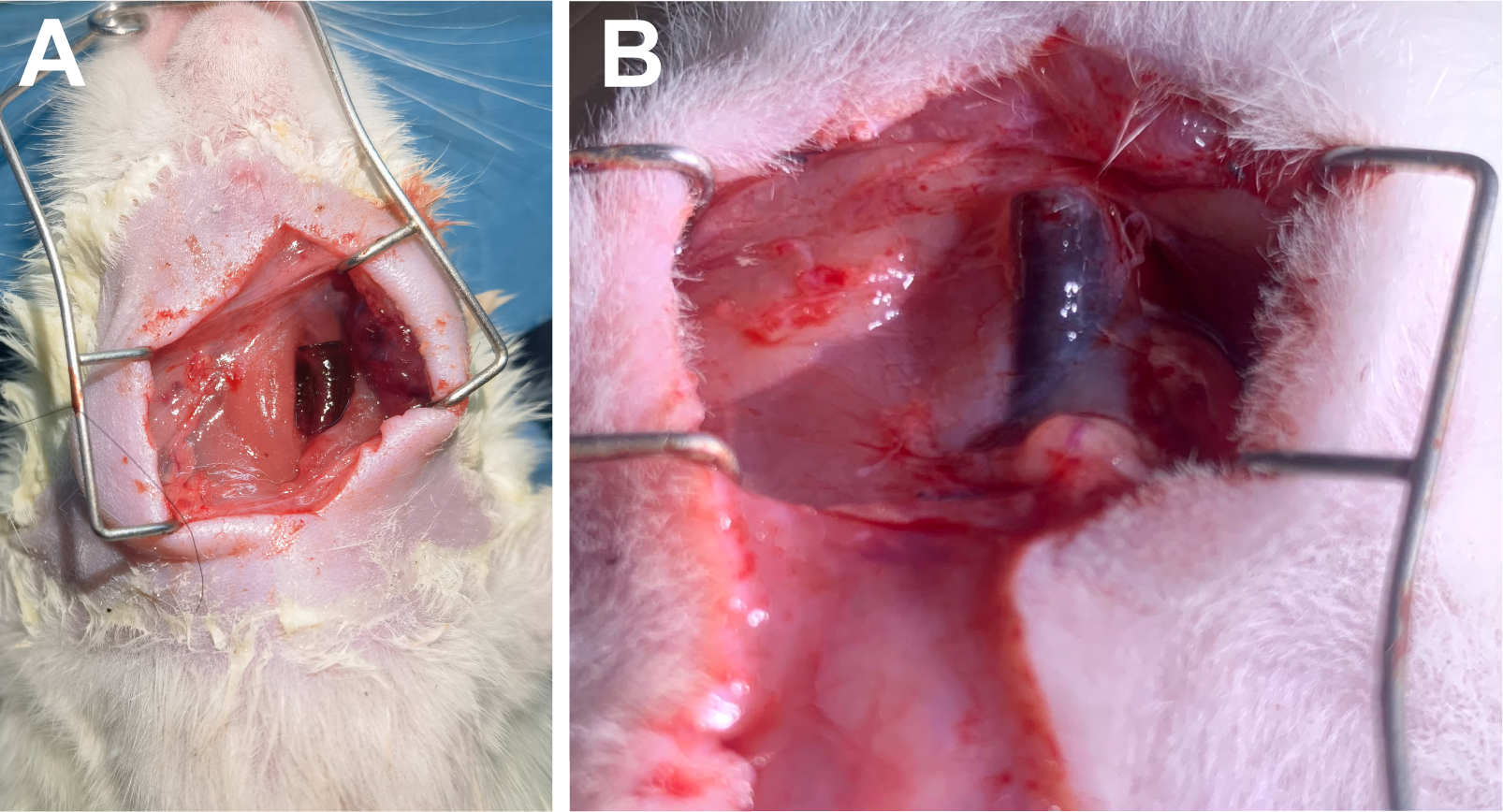

Supplement: Supplementary file 1 [file Image3.TIF]

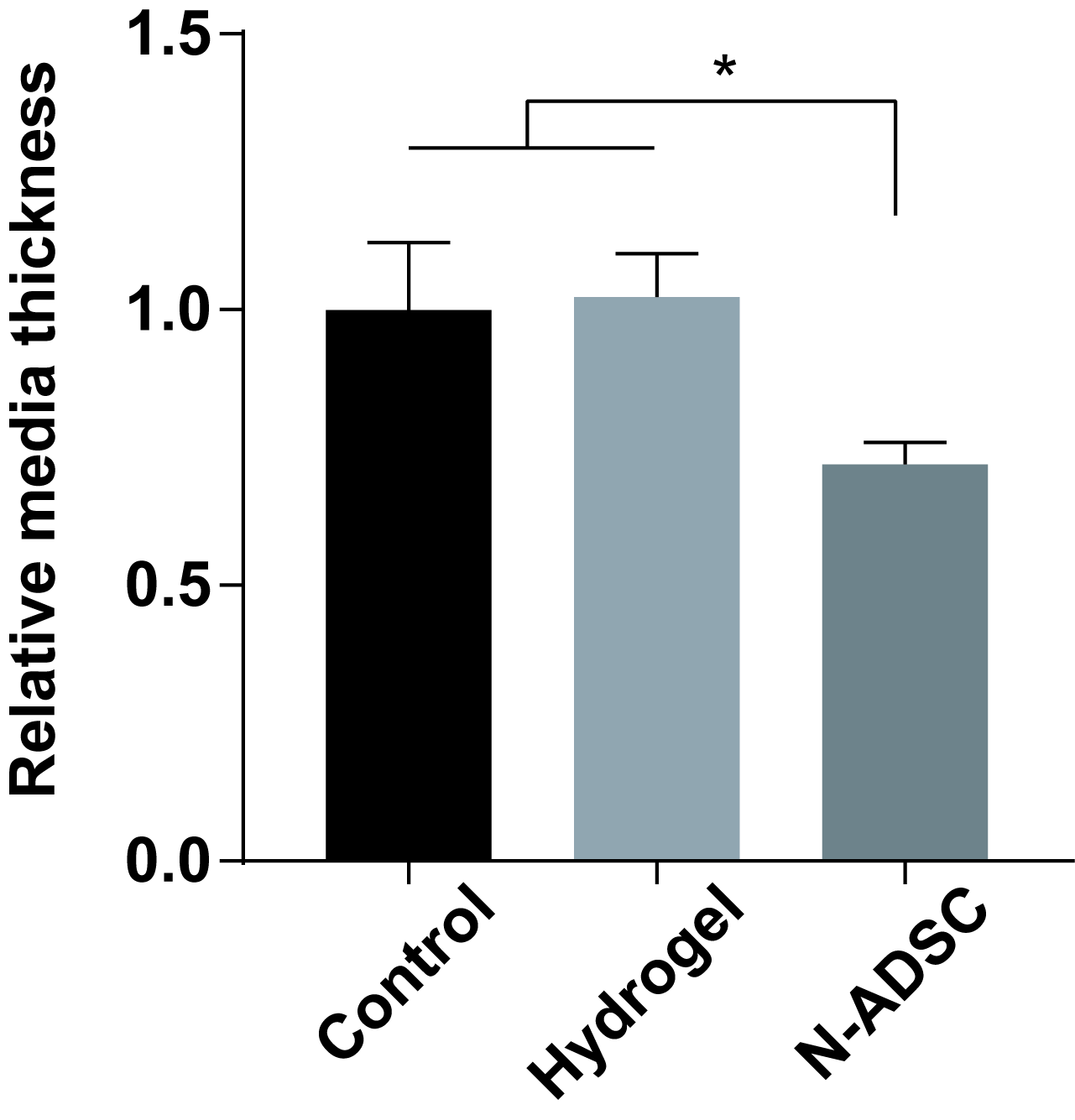

Supplement: Supplementary file 2 [file Image4.TIF]

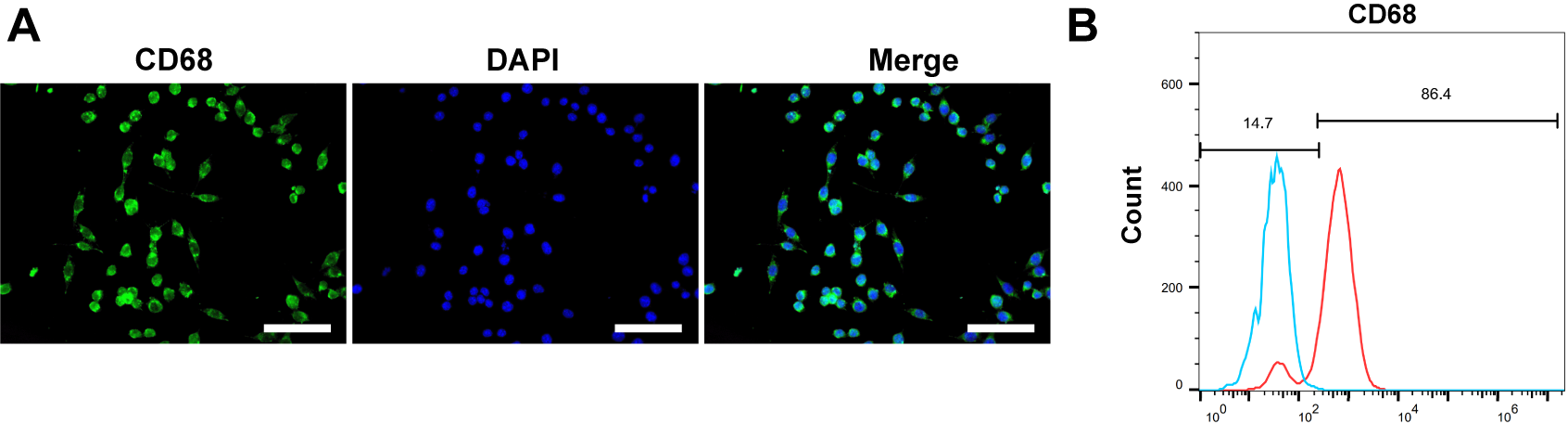

Supplement: Supplementary file 3 [file Image2.TIF]

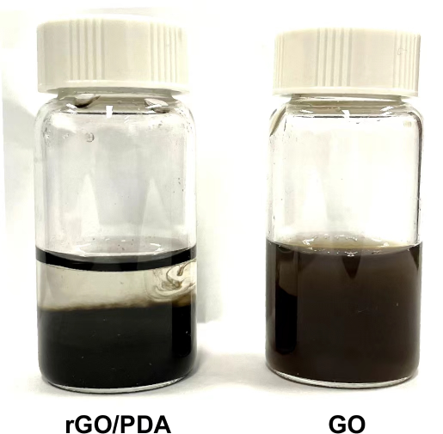

Supplement: Supplementary file 4 [file Image1.TIF]

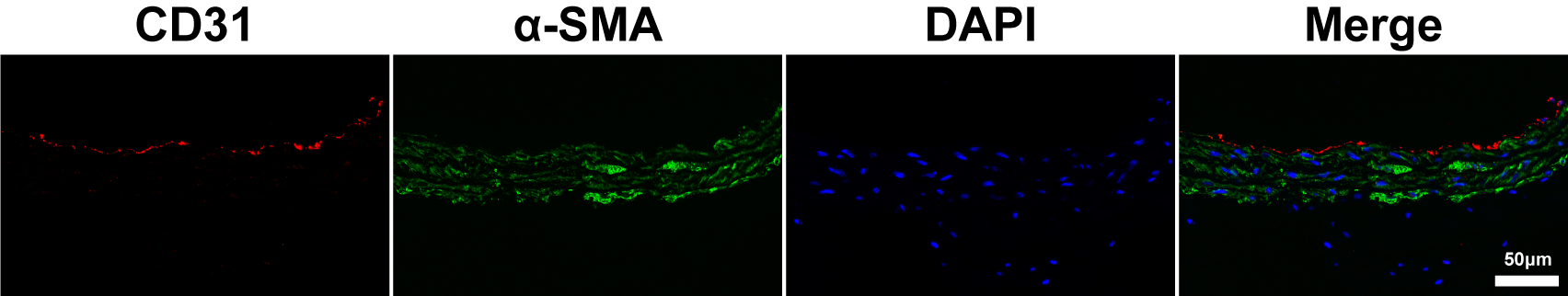

Supplement: Supplementary file 5 [file Image5.TIF]
